# Berg’s Surgical Diagnosis

**Published:** 1906-02

**Authors:** 


BERG'S SURGICAL DIAGNOSIS. A Manual of Surgical Diagnosis. For Students and Practitioners. By Albert A. Berg, M.D., Adjunct Attending Surgeon to Mt. Sinai Hospital, New York. In one 12mo volume of 543 pages with 215 engravings and 21 full page plates. Cloth, $3.25, net. Lea Brothers & Co., Publishers, Philadelphia and New York.

Aseptic surgery and improvements in operative technique in recent years have brought the internal organs within the range of successful treatment and at the same time demanded more accurate methods of diagnosis. Dr. Berg has covered the subject of surgical diagnosis concisely and in its modern development. The diagnosis of kidney diseases from the appearance of the ureteral orifice is discussed.

The engravings are numerous, well executed and all carry a message.

